# Deploying MMEJ using MENdel in precision gene editing applications for gene therapy and functional genomics

**DOI:** 10.1093/nar/gkaa1156

**Published:** 2020-12-10

**Authors:** Gabriel Martínez-Gálvez, Parnal Joshi, Iddo Friedberg, Armando Manduca, Stephen C Ekker

**Affiliations:** Dept. of Physiology & Biomedical Engineering, Mayo Clinic, Rochester, MN, USA; Program in Bioinformatics and Computational Biology, Iowa State University, Ames, IA, USA; Program in Bioinformatics and Computational Biology, Iowa State University, Ames, IA, USA; Dept. of Veterinary Microbiology and Preventive Medicine, Iowa State University, Ames, IA, USA; Dept. of Physiology & Biomedical Engineering, Mayo Clinic, Rochester, MN, USA; Dept. of Biochemistry and Molecular Biology, Mayo Clinic, Rochester, MN 55905, USA

## Abstract

Gene-editing experiments commonly elicit the error-prone non-homologous end joining for DNA double-strand break (DSB) repair. Microhomology-mediated end joining (MMEJ) can generate more predictable outcomes for functional genomic and somatic therapeutic applications. We compared three DSB repair prediction algorithms – MENTHU, inDelphi, and Lindel – in identifying MMEJ-repaired, homogeneous genotypes (PreMAs) in an independent dataset of 5,885 distinct Cas9-mediated mouse embryonic stem cell DSB repair events. MENTHU correctly identified 46% of all PreMAs available, a ∼2- and ∼60-fold sensitivity increase compared to inDelphi and Lindel, respectively. In contrast, only Lindel correctly predicted predominant single-base insertions. We report the new algorithm MENdel, a combination of MENTHU and Lindel, that achieves the most predictive coverage of homogeneous out-of-frame mutations in this large dataset. We then estimated the frequency of Cas9-targetable homogeneous frameshift-inducing DSBs in vertebrate coding regions for gene discovery using MENdel. 47 out of 54 genes (87%) contained at least one early frameshift-inducing DSB and 49 out of 54 (91%) did so when also considering Cas12a-mediated deletions. We suggest that the use of MENdel helps researchers use MMEJ at scale for reverse genetics screenings and with sufficient intra-gene density rates to be viable for nearly all loss-of-function based gene editing therapeutic applications.

## INTRODUCTION

Precision in gene editing is currently limited by the high variability in genotypic outcomes of the commonly deployed NHEJ repair pathway or the low efficiency of the more precise homologous recombination pathway (for reviews, see (1–4)). These shortcomings often result in complicated and labor-intensive selection processes for identifying gene edits of interest, particularly if pursuing bi-allelic editing of vertebrate cells (5,6). When modifying cell lines, for instance, even a high efficiency gene editor can result in one-third of the individual alleles remaining in-frame from NHEJ-based repairs; as a result, knockout cells where all copies contain frameshift alleles represent a minority of outcomes (2,4). Additionally, such molecular heterogeneity makes genotype/phenotype correlation in F_0_ founders of model organisms like zebrafish difficult to interpret, frequently requiring complicated and multi-generational mating schemes to create genotypically homogeneous animals before any direct physiological assays can be performed (5,6). These limitations of NHEJ-based gene editing also potentially reduce its utility in somatic applications such as gene therapy or gene discovery. To address this technical gap in the field, we have developed alternative gene editing approaches that aim to elicit MMEJ (Microhomology-Mediated End Joining) instead of NHEJ and are precise, efficient, and suitable for reverse genetics applications.

Following a DNA double-strand break (DSB), MMEJ is thought to bridge the resulting DNA gap by: annealing a pair of short stretches (3–12 bases) of single-stranded homologies (microhomologies: μHs) exposed by the 5′-resection of the DSB ends (7), trimming the resulting 3′-flap overhangs (8,9), and finally repairing the backbone by DNA ligation (10,11). This process results in a characteristic deletion where the sequence between the pair of μHs used for repair and one of the repeats itself is lost (12) (see (13) for a review). This deletion pattern is useful as a heuristic to identify probable MMEJ-based repairs from a mixed-repair pool. The ability to generate predictable genotypes (14), sometimes even resulting in a single majority outcome (an identical allele in over half of the editing outcomes) (15), makes MMEJ an attractive alternative to NHEJ for precision genome engineering (16–19).

**Figure 1.**
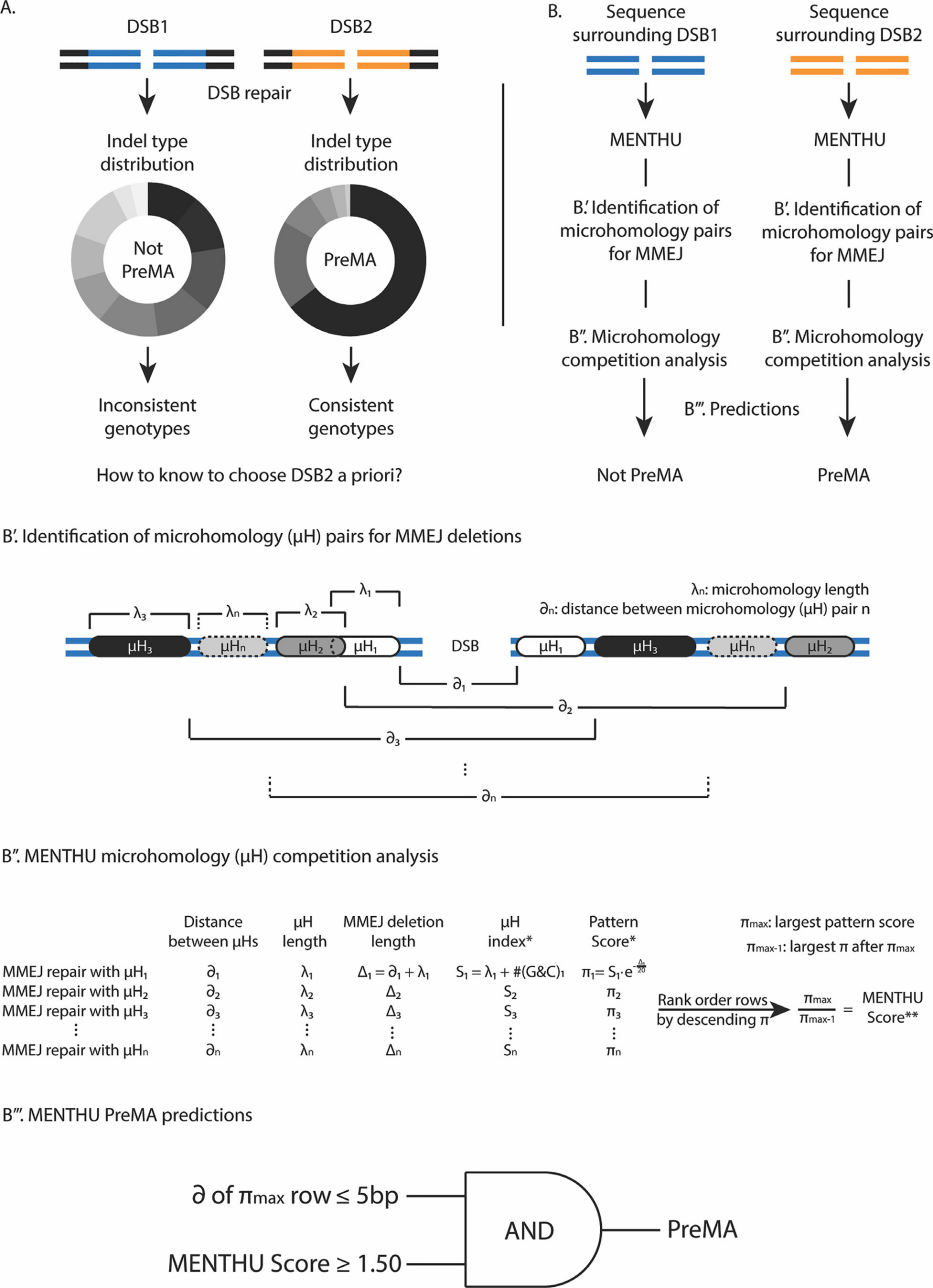
MENTHU predicts which DNA double-strand breaks likely result in single majority deletions. (**A**) Different DNA double-strand breaks (DSBs) can generate indel profiles with dissimilar distributions. Being able to discern the genotype heterogeneity level between targetable DSBs prior to experimental applications would be beneficial for reverse genetics and gene therapy applications. (**B**) MENTHU (22) is a software tool that analyzes the DNA sequence surrounding any given DSB and predicts whether it will result in a PreMA: an MMEJ-mediated repaired sequence where half or more of the repair outcomes share the same genotype. B’. MENTHU identifies every possible μH pair (with homology arms μH_1_ to μH_n_ of length λ_1_ to λ_*n*_) and calculates the corresponding distance between the μHs of each pair (∂_1_ to ∂_n_). B’’. Based on the expected MMEJ deletion pattern, ∂_i_ and λ_i_ are used to calculate the expected deletion length Δ_i_. Pattern scores π_i_ for every possible MMEJ deletion are calculated as described by Bae et al. (16). The MMEJ deletions are then rank ordered by descending pattern score and a MENTHU Score for the DSB is calculated by taking the ratio between the largest πmax and the second largest pattern score π_max-1_. B’’’. MENTHU utilizes two criteria that need to be concomitantly true for a DSB to be labeled as a PreMA. The ∂ of the MMEJ-deletion with the highest pattern score π_max_ and the MENTHU Score for the DSB need to be less than or equal to 5 bp and more than or equal to 1.50, respectively, for a positive PreMA prediction.

We and others (15,16,19) have shown that DSBs directed at sites likely to be repaired via MMEJ significantly increase the homogeneity of the resulting repair outcomes in zebrafish. Despite there being no known clear biochemical mechanism as to how MMEJ repairs DSBs, we and others (20–23) have published software tools that predict the occurrence of MMEJ based only on the sequence context surrounding any given targetable DSB site. In particular, we published MENTHU (22), which screens genetic sequences for DSBs likely to generate single majority outcomes as a result of MMEJ, aka PreMAs (predominant MMEJ alleles) (Figure 1A and B). Opposed to conventional targeting designs, the use of MENTHU-recommended SpCas9 and TALEN cut sites resulted in PreMAs more often, facilitating subsequent zebrafish mutant screenings by decreasing the sequence variability of the resulting allele pool (15). In parallel, the tools ForeCasT (20) and inDelphi (23) were simultaneously and independently developed to predict the probability of occurrence of individual repair outcomes after Cas9-mediated DSBs on mammalian cells, and were shortly followed by Lindel (21). Here, we provide an out-of-sample validation of the PreMA prediction performance of MENTHU in the mouse embryonic stem cell (mESC) dataset used to train ForeCasT, compare it to that of inDelphi and Lindel, and suggest improvements to MENTHU. Additionally, we assess the ability of inDelphi and Lindel to predict single majority frameshifts through 1 bp insertions and consequently propose MENdel, a combination between MENTHU and Lindel to predict DSBs likely to result in frameshift-inducing single majority outcomes. Finally, we assess the practicality of MENdel in a common use-case scenario: the generation of gene knockouts via frameshift mutations. For this, we screened a test set of 54 vertebrate genes (human and zebrafish) of varying sizes for early Cas9 and Cas12a targetable sites likely to result in a single majority frameshift. This would provide an initial estimate on the usefulness of DNA repair prediction tools like MENdel to identify reproducible DSB repair sites based on their frequency and distribution within the coding sequence of genes. We report how MENTHU and inDelphi perform comparably better than Lindel at predicting PreMAs, how Lindel provides the best predictive performance at predicting 1bp insertions, and how MENdel displayed the largest coverage for frameshift predictions across all tools analysed. Thus, we encourage genome engineers to deploy MENdel to design their gene editing experiments for functional genomics or gene therapy, supported by our estimates that ∼90% of vertebrate genes will have at least one early targetable DSB site likely to result in a single majority frameshift.

## MATERIALS AND METHODS

### Sequence data acquisition, inclusion, and classification

**Figure 2.**
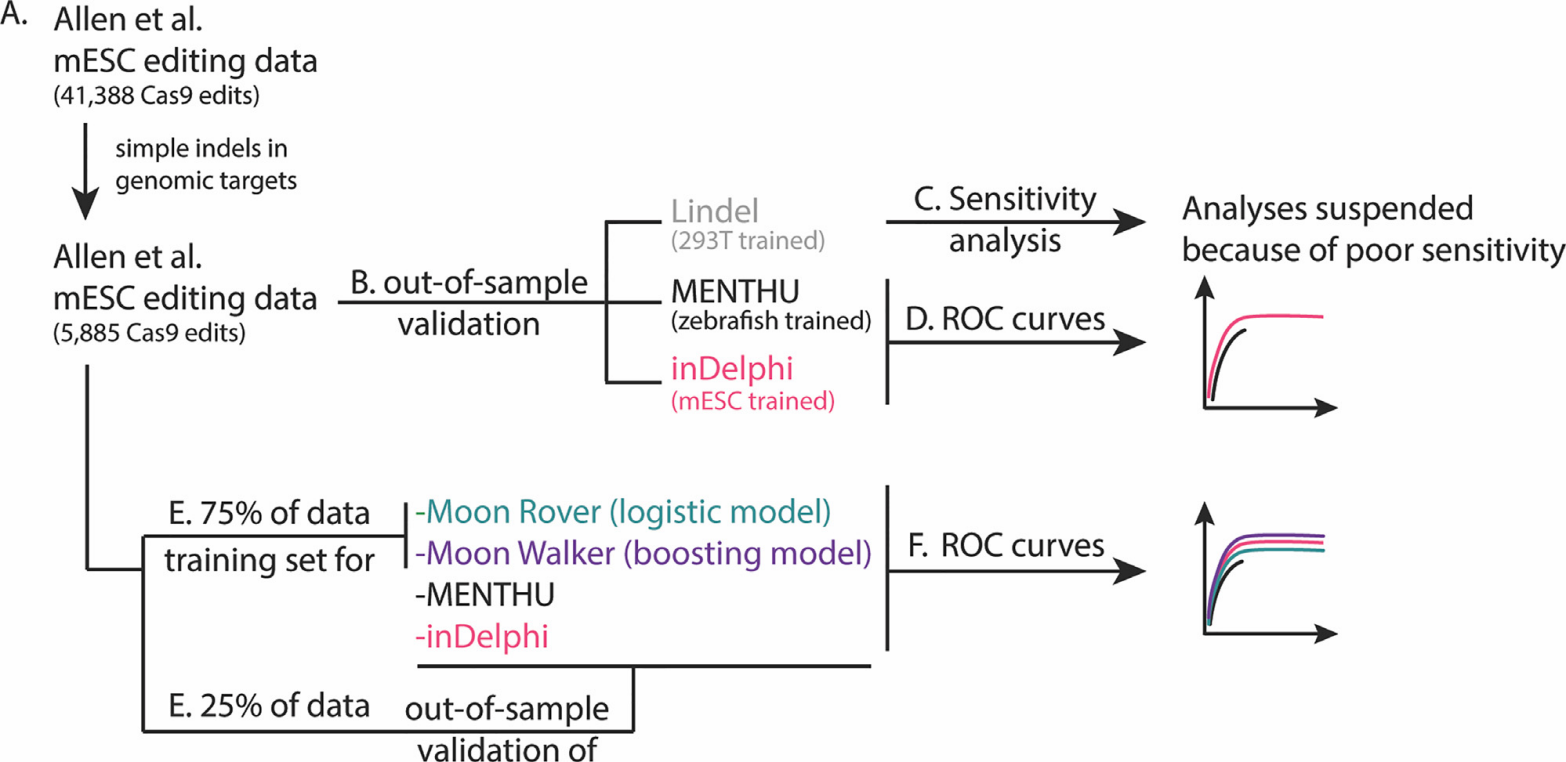
Workflow of the independent assessment of the ability of MENTHU to predict PreMAs. (**A**) A large gene editing dataset was filtered to only include genomic DSB repair outcomes that resulted in simple indels (i.e. resulting in single deletions or insertions). (**B**) This dataset was used to assess the viability of MENTHU PreMA predictions in a mammalian cell system (mouse ESC cells [mESCs]), since MENTHU was originally validated in zebrafish embryos. To contextualize any MENTHU claims, the same dataset was used to generate PreMA predictions using inDelphi and Lindel, similar-purpose software tools in the recent literature. (**C**) Lindel predictions resulted in less than 1% sensitivity and were therefore excluded from downstream PreMA analyses. (**D**) Receiver Operating Characteristic (ROC) curves were used to compare the ability to predict PreMAs by MENTHU and inDelphi. (**E**) To investigate whether the MENTHU prediction scheme maximizes the predictive capacity of the features it uses for classification, the large dataset described in (A) was split into 75% for the training of machine learning models for PreMA predictions and 25% for the out-of-sample evaluation of these models. (**F**) The training set in (E) was used to train Moon Rover (a logistic regression classifier) and Moon Walker (a gradient boosting machine classifier). ROC curves for Moon Rover and Moon Walker were generated based on their predictive performance on the testing set in (E), and were plotted together with ROC curves of MENTHU and inDelphi on the same testing set for reference.

Due to the lack of a comprehensive database for deep sequencing results of DSB repair events, the mESC subset of the data generated by Allen *et al.* (20) to develop ForeCasT was chosen to assess the predictive performance of MENTHU and compare it to inDelphi and Lindel predictions. ForeCasT (20) was excluded from these comparisons to avoid training bias. This dataset is ideal for an unbiased comparison between MENTHU, inDelphi, and Lindel since it is the largest available source of DSB repair data not used to train any of the tools being compared. A total of 41,388 different Cas9-mediated DSB repair events in mESC cells were downloaded from https://figshare.com/articles/processed_mutational_profiles/7312067. For each event, all resulting repair sequences from all experimental replicates were combined into a single pool, consolidated by sequence, and rank ordered by number of reads. Subsequently, the most frequently observed read was aligned to its corresponding WT sequence (obtained from the Supplementary Data 1 at (20)) using the pairwiseAlignment function from the Bioconductor Biostrings package (version 2.54.0) with a substitution matrix that heavily penalized mismatches (match = 1, mismatch = –50). Since MENTHU and both inDelphi and Lindel were trained to predict, respectively, single-deletion and single-indel (insertion or deletion) repairs exclusively, only those alignments that could be explained by a single, simple indel were included in the analysis. Each alignment was classified into one of four different groups based on the nature of the observed indel: a 1 bp or >1 bp insertion, or an MMEJ or non-MMEJ deletion. MMEJ deletions were defined as those that displayed, in the WT sequence, two μHs of at least 3 bp in length at each side of the expected DSB, which later collapsed into one in the repaired read deleting any intervening nucleotides. Deletions that did not follow this pattern were considered non-MMEJ deletions. Lastly, to ensure that the results of this study are representative of genome-targeting experiments, any DSB event that employed a gRNAs targeting an artificially manufactured sequence was excluded from the any downstream analyses. This process culminated in a total of 5,885 Cas9-mediated edits, each with its WT sequence, most frequent repair sequence, and corresponding observed frequency (Figure 2A).

### Comparison between PreMA predictive performance of MENTHU, inDelphi and Lindel

For each for the 5,885 Cas9-mediated DSB repair events, a 52bp sequence window centered at the Cas9 expected DSB location (i.e. 3 bases upstream of NGG PAM) was extracted. These short sequences served as inputs for the command-line versions of MENTHU (R) and inDelphi (Python 2.7) to generate PreMA predictions. Lindel (Python 3.7) requires 60 bp of sequence context for predictions so the sequence window was adjusted accordingly. For every input, MENTHU outputs a data frame with all possible MMEJ-based deletions within the 52bp sequence (Figure 1B’ and B’’) and rank orders them by MENTHU score, as described in Mann et al. (22). We classified MENTHU predictions as recommended by Ata *et al.* (15), labelling as PreMA any site displaying (a) a MENTHU score of 1.50 or above and (b) a distance of 5bp or less in the WT sequence between the μHs utilized for MMEJ of the most frequent predicted repair outcome (Figure 1B’’’). On the other hand, inDelphi and Lindel output the probability of occurrence of the list of potential repair outcomes for every input. Consequently, any inDelphi or Lindel prediction where the most likely sequence outcome has a probability of 0.50 or more and displays the MMEJ deletion pattern was classified as a PreMA. PreMA predictions from MENTHU, inDelphi, and Lindel were compared to their corresponding ground truth sequencing data (Figure 2B) and classified as true positives (TP, when a PreMA prediction matched the data), true negatives (TN, when a non-PreMA prediction matched the data), false positives (FP, when a PreMA prediction did not match the data), and false negatives (FN, when a non-PreMA prediction did not match the data). Sensitivity ($\frac{{TP}}{{TP + FN}}$: the percentage of actual PreMAs correctly classified as such), specificity ($\frac{{TN}}{{TN + FP}}$: the percentage of actual non-PreMAs correctly classified as such), and positive predictive value ($\frac{{TP}}{{TP + FP}}$: PPV, percentage of correct positive PreMA predictions) were calculated for all tools. Of note, Lindel was excluded from further PreMA analyses because of its poor sensitivity when compared to MENTHU and inDelphi. (Figure 2C). Additionally, the possibility of utilizing MENTHU and inDelphi synergistically was assessed by calculating sensitivity, specificity, and PPV of the predictions generated by (a) labelling a DSB repair event as a PreMA either when they both did (AND), or (b) when either one of them predicted a PreMA individually (OR).

### Generation of Receiver Operating Characteristic curves for MENTHU and inDelphi

Receiver Operating Characteristic (ROC) curves are a standard technique for evaluating binary classifiers (24), and are plots of TP rate (sensitivity) versus FP rate (1 – specificity, also known as the significance level α) as a function of varying classification thresholds. ROC curves were generated for MENTHU and inDelphi (Figure 2D) to evaluate their predictive performance independent of any specific prediction threshold values (i.e. MENTHU score and inDelphi probability). The MENTHU ROC curve was generated by varying the MENTHU score classification threshold from 0 to infinity while leaving the μH distance requirement (less than or equal to 5bp) constant. ROC curves for inDelphi were generated by varying the predicted probability threshold used for PreMA classifications from 0 to 1 (originally 0.50).

### Development of MENTHU-based PreMA prediction models

The original MENTHU, as described by Ata *et al.* (15), is a threshold-based, two-feature PreMA prediction scheme (Figure 1B’’’). To investigate the impact of the distance threshold component and complement the ROC curve analysis described above, we calculated the sensitivity, specificity, and PPV values of PreMA predictions at 3 other threshold values (less than or equal to 3, 4, and 6bp: MENTHU@3, MENTHU@4, and MENTHU@6, respectively) while keeping the MENTHU score threshold constant. Additionally, we examined the impact of combining the prediction outcomes of the original MENTHU, MENTHU@4, and inDelphi.

The dataset from Allen *et al.* (20) was also used to train and test two machine learning models, Moon Rover and Moon Walker, that employ the same features MENTHU does to predict PreMAs (i.e. the MENTHU score and the distance between the μH pair for the top predicted MMEJ-based outcome). Significant improvements of either of these models over the original MENTHU would suggest a better way to utilize these two features to improve predictive performance. The 5,885 DSB repair events from Allen *et al.* (20) were divided into a training set and a test set in a 70–30% split (4,120 and 1,765 respectively), with the PreMA to non-PreMA ratio remaining constant in both sets (Figure 2E). Moon Rover, a logistic regression model, and Moon Walker, a gradient boosting model (25) were both trained with the same training set to output a binary response (PreMA or no PreMA). Moon Walker used a 10-fold cross validation scheme to choose the set of model hyperparameters that displayed the highest ROC curve area under the curve. Each hyperparameter set was defined by a grid-search of the number of boosting iterations (decision trees), maximum tree depth, minimum amount of observations per node, and shrinkage level (regularization constant). The model performance was assessed by making out-of-sample predictions on the test set. MENTHU and inDelphi ROC curves on the test set were also calculated for comparison (Figure 2E and F). Both models were trained and evaluated using the R-based package caret (26).

### Evaluation of insertion-based single majority outcomes using inDelphi and Lindel and the development of an algorithmic workflow to predict single majority frameshifts

The prediction of single majority outcomes that result from insertions are outside of the design scope of MENTHU. Nonetheless, DSB repair likely to result in insertions, such as 1bp insertions, could be a valuable source of frameshift mutations as an alternative to PreMAs. Consequently, we determined the amount of out-of-frame single majority insertions in the Allen *et al.* DSB repair dataset (20), and proceeded to evaluate the ability of inDelphi and Lindel to predict them. We compared the predicted and observed insertion-based single majority repairs by inDelphi and Lindel and calculated their respective sensitivity, specificity, and PPV as described above for the PreMA predictions. Based on the observed PreMA and 1bp-insertion predictive performances by MENTHU, inDelphi and Lindel, we designed a workflow between these that maximized our ability to predict the single majority frameshifts present in the Allen *et al.* (20) dataset.

### Assessment of PreMA targeting for out-of-frame mutations

Accurate prediction of PreMAs would be of limited value if their frequency or general localization within a gene were not useful experimentally. Therefore, we investigated the intragenic density and localization of PreMAs in 28 human and 26 zebrafish genes (Supplemental Table S1) that were likely to result in early out-of-frame mutations. Using MENTHU, we screened the first 30% of the cDNA sequence of each gene for potential frameshift-resulting Cas9 and Cas12a-mediated PreMAs. To ensure screening of at least 150 bp per cDNA, the first 182bp were screened for shorter coding sequences (150 + 32 bp upstream of DSB site to ensure sufficient sequence context for prediction).

## RESULTS

### Data inclusion

Following the inclusion/exclusion criteria described in the methods section, we extracted a set of 5,885 Cas9-mediated DSB repair events from the Allen *et al.* data (20). Briefly, all of these were required to utilize gRNAs that target known human genomic sequences and have their most frequent observed mutation outcome be a simple indel (Figure 2A). The alignments between the WT sequence and the most frequently observed indel at each individual DSB repair event, constituted ∼54% non-MMEJ deletions, 31% MMEJ deletions, 14% 1 bp insertions, and 0.2% + 1 bp insertions.

### Comparison between PreMA predictive performance of MENTHU, inDelphi and Lindel

**Figure 3.**
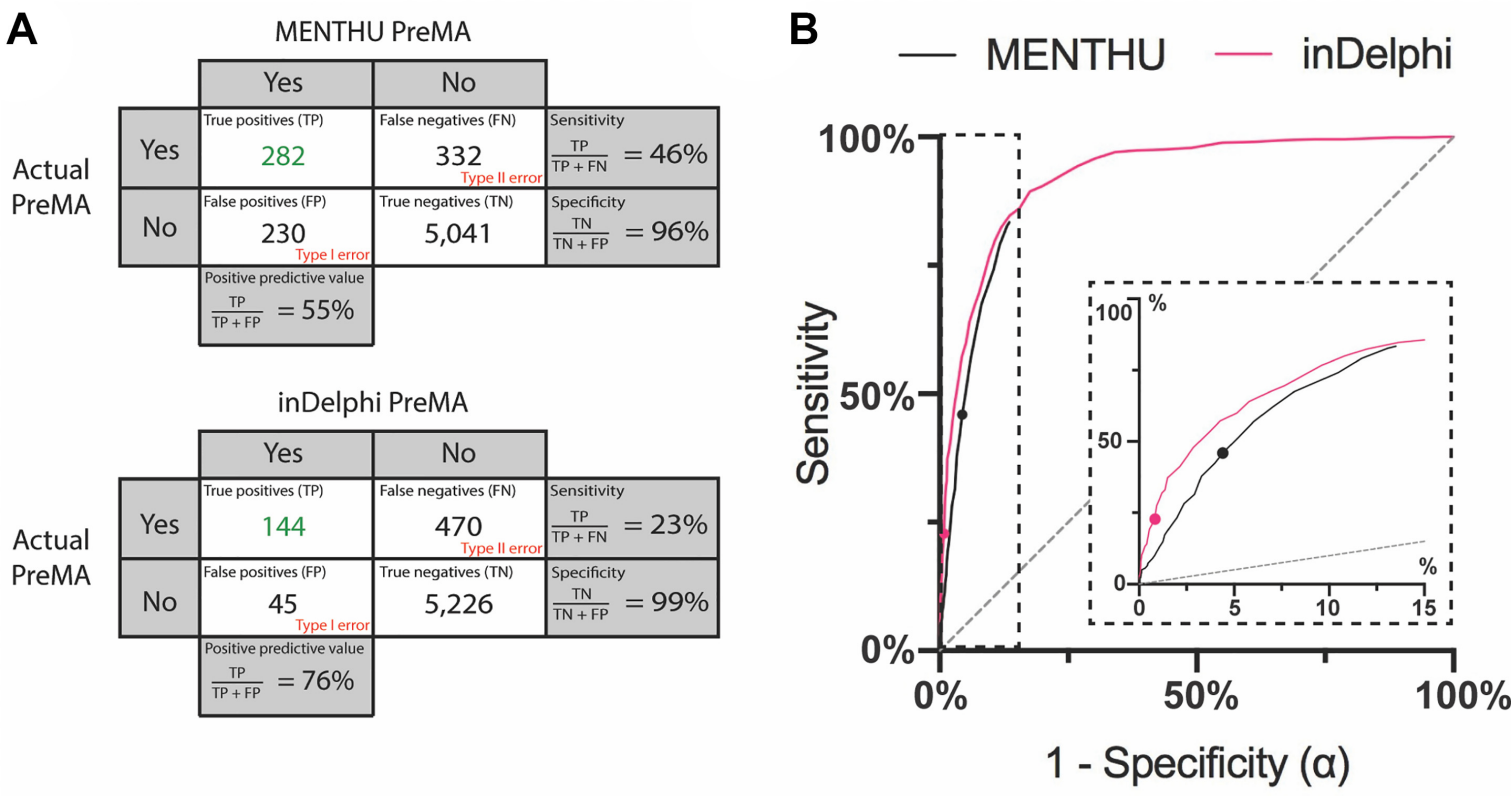
Comparison of the performance of the published versions of MENTHU and inDelphi in predicting PreMAs in a large, out-of-sample dataset. (**A**) Confusion matrices for PreMA predictions by MENTHU (top) and inDelphi (bottom). Rows indicate the PreMA status of 5,885 Cas9 generated mutation profiles in mESC cells taken from Allen *et al.* (20). Columns denote the PreMA predictions by MENTHU and inDelphi. Sensitivity is the proportion of positive-PreMAs correctly predicted as such. Specificity is the proportion of negative-PreMAs correctly predicted as such. PPV is the proportion of correct predictions of positive-PreMAs. (**B**) Receiver Operating Characteristic (ROC) curves comparing MENTHU and inDelphi PreMA predictions. Here, sensitivity is plotted against 1 – specificity (or the probability of a type I error: α) as a function of varying prediction thresholds. The two plotted points represent the published thresholds for both tools. The MENTHU ROC curve was generated by varying the MENTHU score threshold for PreMA classification. In the inDelphi ROC curve, the minimum threshold probability of the most frequent predicted read was varied. The MENTHU curve is truncated because its second classification criterion regarding the maximum distance between μHs allowed for MMEJ classification does not allow for a higher sensitivity. The inset is a blowup of the region where MENTHU is present.

Sensitivity, specificity, and positive predictive value (PPV) were calculated for the PreMA predictions of MENTHU, inDelphi, and Lindel at each of the 5,885 Cas9-mediated DSBs. The corresponding confusion matrices are shown in Figure 3A. Of the 614 PreMAs in the data, MENTHU correctly identified twice the total number as inDelphi (with sensitivities of 46% and 23%, respectively), although with a lower correct classification-rate of PreMA-positive events (55% to 76% PPV, respectively) and slightly lower specificity (96% and 99%, respectively). Lindel was excluded from further PreMA deletion analyses because of its relatively poor sensitivity (0.8%). In terms of PreMA coverage, 27.0% of all available PreMAs were uniquely predicted by MENTHU, 4.6% by inDelphi only, and 18.9% by both (166, 28, and 116 out of 614, respectively). In contrast, the majority (95.3%) of the 5271 non-PreMAs in the data were correctly classified by both tools. Further analysis into these differences revealed that 10/28 of the PreMAs found by inDelphi but not MENTHU failed the MENTHU μH proximity criterion (see ∂ at Figure 1B’ and ‘Sequence data acquisition, inclusion, and classification’ and ‘Sequence data acquisition, inclusion, and classification’ in Materials and Methods), and the remaining 18/28 failed the MENTHU Score 1.50 cutoff (four of them by 0.01 or less).

PreMAs, by definition, appear at a frequency of at least 50% in the mixed repair pool following a DSB repair event. Breaking down the MENTHU false-positive PreMA predictions revealed that over 60% of them failed to reach the ≥50% frequency requirement, albeit displaying the characteristic MMEJ deletion pattern. Over half this subset had >40% frequency, with most displaying a frequency >46%. Importantly, the vast majority of these false-positives MMEJ outcomes (97%) still displayed the exact sequence changes predicted by MENTHU. This finding was consistent across true positives by both MENTHU and inDelphi where, respectively, 100% and 99% of the sequence predictions matched the observed predominant sequence.

### Receiver Operating Characteristic (ROC) curves for MENTHU and inDelphi predictions

ROC curves allow for the comparison between predictive algorithms regardless of the thresholds ultimately chosen for prediction. In general, the point in the curve closest to the top-left coordinate (1,0) represents the prediction threshold that maximizes sensitivity and specificity. However, the prediction threshold to use should ultimately be decided on a case-by-case basis. The ROC curves for MENTHU and inDelphi are shown in Figure 3B. The MENTHU ROC curve is truncated because MENTHU classifies any MMEJ prediction that does not comply with the maximum of 5bp distance between μHs as a non-PreMA. Hence, the maximum achievable sensitivity (the top-left most point of the ROC curve) was 83.39%.

### Refinement of MENTHU-based PreMA prediction models

**Figure 4.**
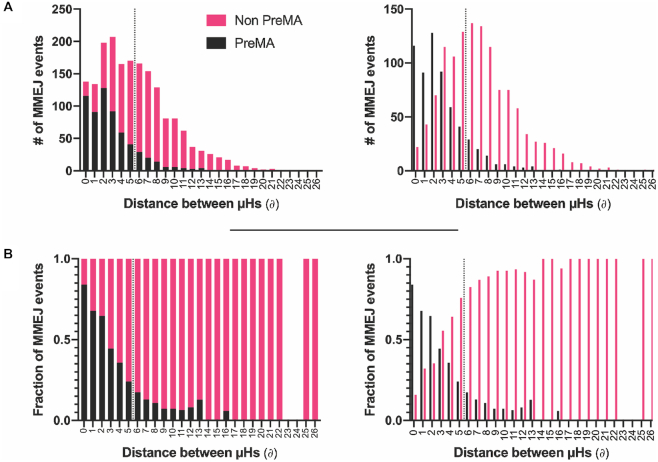
PreMA distribution of MMEJ events as a function of the distance between the microhomologies employed for repair. (**A**) Stacked (left) and staggered (right) distributions of the number of MMEJ repair events in a large gene editing data set (20) and their PreMA status were plotted as a function of the distance between the microhomologies (μHs) used for repair (∂). The amount of MMEJ events increases after a ∂ of 1 bp and then decreases consistently as a function of ∂ after 5 bp. (**B**) The fraction of PreMAs across each ∂ bin in A is plotted as a function of ∂. The PreMA fraction decreases in an exponential-like fashion as a function of ∂. The dotted lines in both A and B represent the classification threshold employed by MENTHU for PreMA predictions. Everything to the left of the dotted line is predicted as PreMA as long as the corresponding MENTHU score is ≥1.50.

As evidenced by the ROC curves described above, choosing different MENTHU score thresholds results in trade-offs between sensitivity and specificity. Since MENTHU classifications rely on two different thresholds (Figure 1B’’’), we looked at varying the distance threshold (∂) while keeping the MENTHU score threshold constant to observe changes in the PreMA predictive performance (Table 1). Figure 4A shows how all 1844 MMEJ repaired events in the data, PreMA and not PreMA, are distributed as a function of ∂. Figure 4B displays the distribution of PreMAs across each bin of Figure 4A. We found that, on this data set, MENTHU increased its PPV and specificity to ∼65% and 97% (∼10% and a ∼1.5% increase, respectively) by decreasing the ∂ by 1bp (to 4 bp) in exchange for a ∼4.5% sensitivity loss (MENTHU@4). We also looked at whether combining the MENTHUs and/or inDelphi PreMA predictions resulted in a better performance (Table 2). The best combination was MENTHU@4 or inDelphi (i.e. predict a PreMA if either algorithm makes this prediction), achieving a 64% PPV and a ∼1% increase in both sensitivity and specificity in comparison to the original MENTHU.

**Table 1.** Summary of the PreMA predictive performance of MENTHU at different μH distance thresholds in bp (@x bp)

*Model*	Sensitivity	Specificity	PPV
*MENTHU@3*	36.64%	97.99%	67.98%
*MENTHU@4*	41.53%	97.36%	64.72%
*MENTHU@5	45.93%	95.64%	55.08%
*MENTHU@6*	48.70%	93.54%	47.16%

*MENTHU@5 represents the performance metrics by the original MENTHU. PPV: Positive predictive value, the percentage of correct positive PreMA predictions.

**Table 2.** Summary of the PreMA predictive performance of different combinations between MENTHU and inDelphi

*Model combination*	Sensitivity	Specificity	PPV
*MENTHU alone*	45.93%	95.64%	55.08%
*MENTHU@4 alone*	41.53%	97.36%	64.72%
*inDelphi alone*	23.45%	99.15%	76.19%
*MENTHU or inDelphi*	50.49%	95.28%	55.46%
*MENTHU and inDelphi*	18.89%	99.51%	81.69%
*MENTHU@4 or inDelphi*	46.91%	96.95%	64.14%
*MENTHU@4 and inDelphi*	18.08%	99.56%	82.84%

PPV: positive predictive value, the percentage of correct positive PreMA predictions.

To investigate whether the two features that MENTHU utilizes for PreMA predictions can be numerically optimized, two machine learning algorithms (Moon Rover and Moon Walker) were developed using the same inputs and outputs as MENTHU.

**Figure 5.**
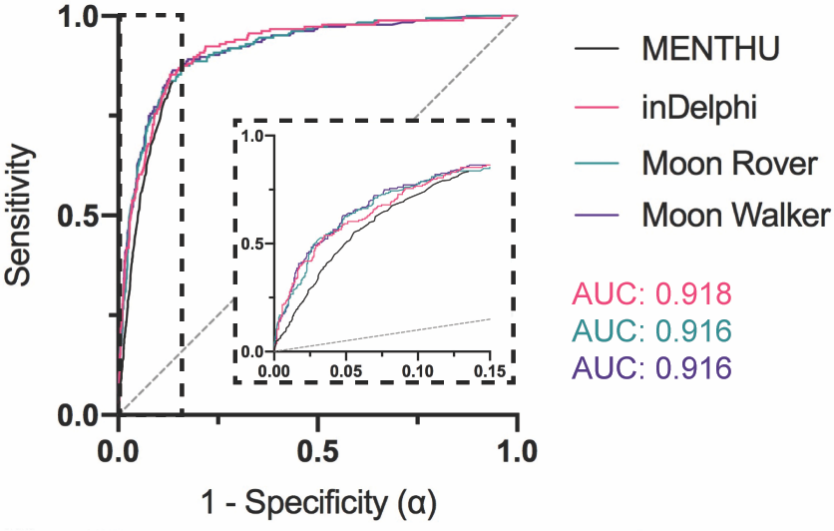
Receiver Operating Characteristic (ROC) curves comparing the prediction performance of MENTHU and inDelphi to that of the novel MENTHU-based tools Moon Rover and Moon Walker. Moon Walker and Moon Rover are two machine-learning-based tools that utilize the same two features for PreMA predictions that MENTHU uses: the MENTHU Score and the distance between the microhomologies used for most expected MMEJ repair outcome. The ROC curves displayed represent the PreMA prediction performance of MENTHU, inDelphi, Moon Rover, and Moon Walker on the out-of-sample validation set described on Figure 2E. Here, sensitivity is plotted against 1 – specificity (or the probability of a type I error: α) as a function of varying prediction thresholds. See Figure 3 legend for explanation on MENTHU and inDelphi thresholds. The inset is a blowup of the region where MENTHU is present. The area under the curve for inDelphi, Moon Rover, and Moon Walker are 0.918, 0.916 and 0.916, respectively.

- *Moon Rover*. A logistic regression model with MENTHU score and ∂ as inputs and a binary PreMA/non-PreMA classification as the output.
- *Moon Walker*. A gradient boosting model (25) based on decision trees (27) was trained on the same dataset as Moon Rover using the same input/output scheme. The hyperparameter combination that yielded maximum ROC area under the curve (AUC) utilized 450 trees with six levels of interaction depth, a minimum of 10 observations per node and a 0.01 shrinkage. ROC curves for the PreMA prediction performance on the test set by MENTHU, inDelphi, Moon Rover, and Moon Walker were generated (Figure 5). Moon Rover and Moon Walker each showed small AUC improvements over MENTHU, similar to inDelphi.

### Estimation of PreMA frequency and distribution in vertebrate genomes

To assess the usefulness of PreMA targeting for reverse genetics applications, we investigated the frequency and localization of MENTHU-predicted PreMAs across 54 (28 human, 26 zebrafish) genes. The estimated PreMA frequency was consistent with previous reports (15,23), amounting to ∼10% of all targetable sites for both human and zebrafish. As expected, Cas12a, a nuclease with more targeting constraints than Cas9 (TTTV vs NGG) (28), displayed fewer potential knockout-inducing PreMAs. We also found that, when considering Cas9 alone (i.e. NGG PAMs), 81% of the genes screened (44 out of 54) have at least one predicted PreMA site in the first 30% of their coding sequences. This number increases to 87% (47/54) when also considering Cas12a.

### Evaluation of insertion-based predominant sites and their prediction by inDelphi and Lindel

To estimate the need for insertion-based single majority outcome predictions, which MENTHU is unable to generate, we determined the amount of insertions in the Allen *et al.* data (20) relative to PreMAs. More specifically, we were interested in assessing the added benefit of using inDelphi or Lindel to predict single majority insertions that would cause frameshift mutations. Out of the 5,885 observed DSB repair events, 839 (∼14%) resulted in insertions. Of these, 826 (∼98%) were 1bp insertions, of which only 186 resulted in single majority outcomes. While inDelphi failed to predict any insertion-based single majority outcomes, Lindel correctly identified 62 out of the 186, amounting to a sensitivity of 33%, a specificity of 99%, and a 60% PPV by incurring in 42 false positives.

### MENdel: deploying MENTHU and Lindel together provides the most predictive coverage for out-of-frame mutations

**Figure 6.**
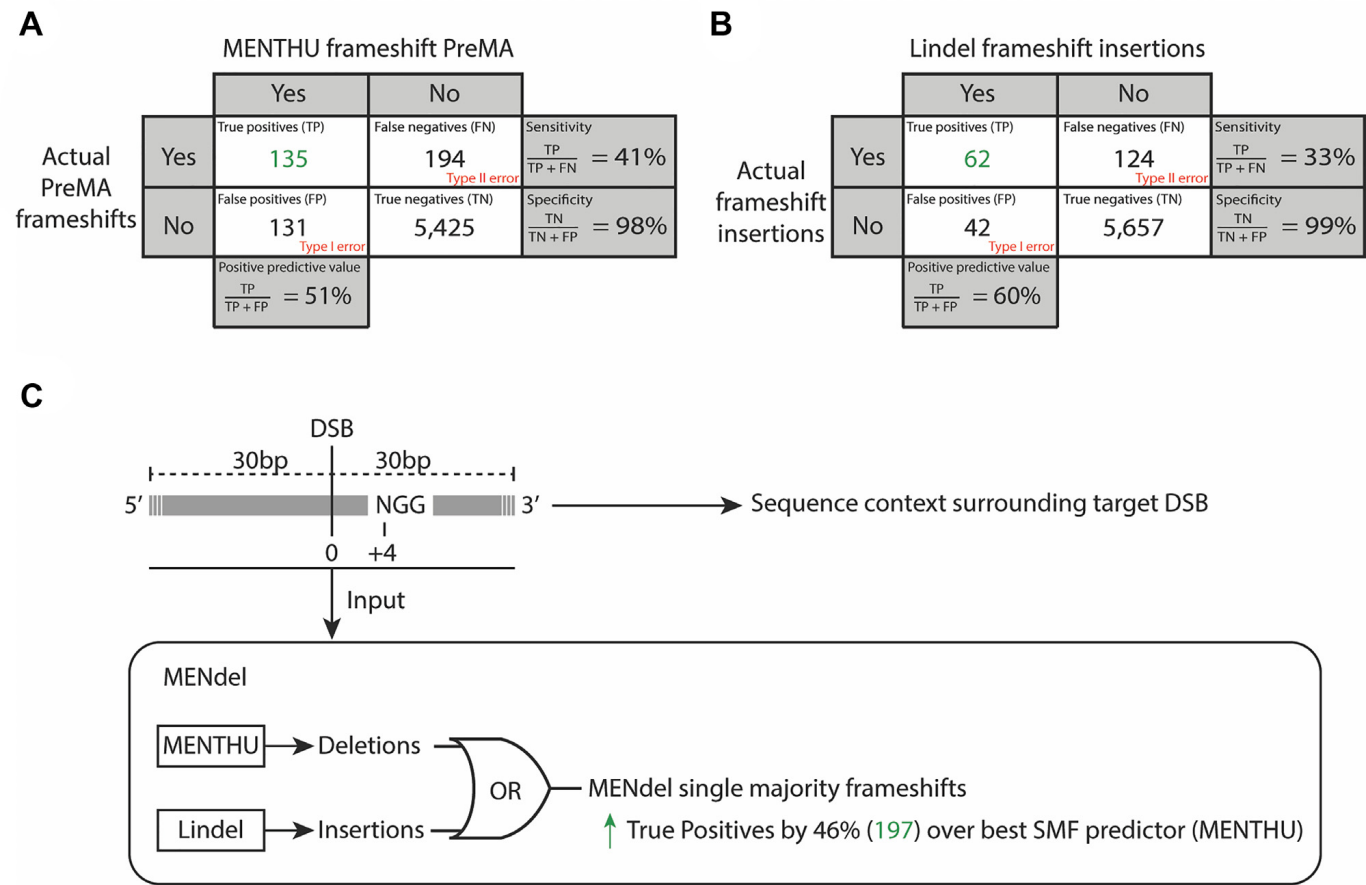
MENdel predicts which DNA double-strand breaks likely result in single majority deletions and insertions for likely frameshift loss of function alleles. The confusion matrices display the performance of the prediction of (**A**) frameshift-inducing PreMAs by MENTHU and (**B**) insertion frameshifts by Lindel across all 5,885 Cas9-mediated edits from Allen *et al.* (20). (**C**) MENdel takes 60bp of sequence context centered at a SpCas9-targetable DSB site to predict single majority deletions (PreMAs) using MENTHU and single majority insertions using Lindel. MENdel offers ∼46% more true-positives of frameshift alleles (197) than MENTHU alone (135).

In short, while MENTHU and inDelphi possess distinct advantages over each other for PreMA prediction (where Lindel performed comparably poorly), MENTHU offers more prediction flexibility by allowing users to accommodate prediction thresholds. In contrast, Lindel outperformed inDelphi at identifying single majority insertions, something MENTHU was not designed to do. Consequently, we constructed MENdel, a workflow that combines MENTHU and Lindel to predict DSB sites likely to result in out-of-frame single majority outcomes either by MMEJ deletions or 1 bp insertions (Figure 6). MENTHU alone was able to correctly identify 135 out of the 329 frameshift PreMAs in the data. This corresponds to 44%, 98% and 51% sensitivity, specificity, and PPV, respectively; consistent statistics to the overall MENTHU PreMA predictive performance. Like before, the inDelphi sensitivity to frameshift PreMAs (17%) was lower than that of MENTHU. By ignoring any Lindel PreMA predictions and focusing only on its insertion predictions, MENdel adds these out-of-frame insertion predictions on top of the MENTHU frameshift PreMA predictions, correctly identifying 197 of 318 total out-of-frame MMEJ- or insertion-based frameshifts in the data (169 false positives). Thus, MENdel provides a larger predictive coverage of frameshift mutations without any significant performance sacrifices, displaying similar prediction metrics to MENTHU alone (41% sensitivity, 97% specificity, 54% PPV). MENdel was also used to screen the first 30% of the coding sequences of 54 vertebrate genes for DSB sites likely to result in single majority frameshifts via PreMAs or 1bp insertions. MENdel found that 47 out of the 54 genes (87%) screened possess at least one frameshift-inducing Cas9-targetable site likely to result in a single majority outcome.

## DISCUSSION

**Figure 7.**
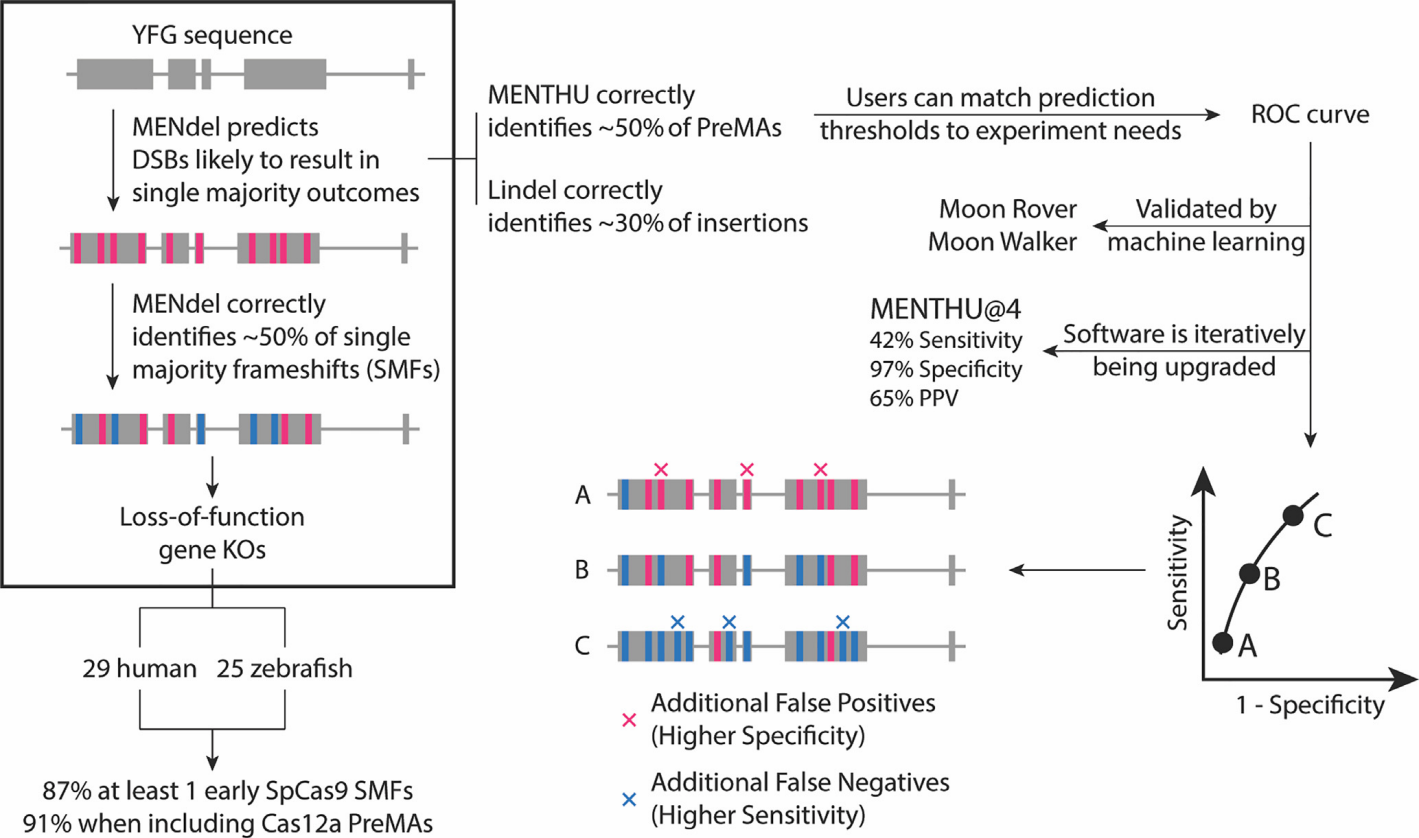
MMEJ-targeting of double-strand break sites for functional genomics and gene therapies. MENdel provides genome engineers with the largest prediction coverage of single majority frameshifts for loss-of-function experiment design (boxed). We sampled 54 vertebrate genes for knockout-generating PreMAs using MENTHU and MENdel, and estimated that the majority (∼90%) of vertebrate genes should possess at least one early out-of-frame single majority outcome. MENTHU (right) is the only double-strand break repair prediction algorithm that allows DNA targeting with nucleases different to SpCas9 and offers scientists with customizable prediction thresholds to best accommodate user needs.

The success of gene editing applications from gene discovery to gene therapy is critically dependent on the specific sequence changes made at each genetic locus. For example, different outcomes due to as little as a single base difference have the potential to substantially alter the observed phenotype in gene therapy uses (e.g. in-frame versus frameshift alleles). Similarly, a failure to generate a true loss-of-function allele could yield a false negative result for gene discovery testing. Gene editing today largely underappreciates the inherent limitations of using NHEJ for the generation of diploid knockouts. Even with 100% efficiency cutting, NHEJ will produce a frameshift two thirds of the time by random chance. Assuming that the repair of both chromosomal copies of the target side are independent events, dual-allele knockouts would occur at an expected frequency <50% $( {\frac{2}{3} \times \;\frac{2}{3} = \frac{4}{9}\;} )$. This represents an upper efficiency limit for the generation of loss-of-function alleles where a gene editing outcome cannot be selectively enriched for (clonal expansion of a cell or germline propagation of an animal). Conversely, for somatic genetic therapeutics or for somatic loss of function science in animal models, MMEJ has the potential to result in up to 100% of dual loss-of-function alleles. In addition, MMEJ alleles provide editing reproducibility to the nucleotide level, which NHEJ alleles cannot. This is beneficial for the reproducibility of gene editing applications able to be clonally expanded or propagated. The goal of this study was to validate MENTHU as an MMEJ-based gene editing tool on an independent dataset and to utilize gene editing outcome data to generate a new prediction tool MENdel for improvements in gene editing precision applications (Figure 7).

We have highlighted how biasing DNA repair mechanisms towards MMEJ reduces the heterogeneity of gene editing outcomes resulting from the more common NHEJ repair pathway, thus offering important advantages for reverse genetics and therapeutic applications. Here we estimated the usefulness of computational tools MENTHU, inDelphi, and Lindel to identify DSBs likely to preferentially undergo MMEJ repair (PreMAs; Figure 1B) in a large independent dataset. To assess the generalizability of MENTHU results beyond the *in vivo* work in zebrafish (15), we measured the ability of MENTHU to predict PreMAs in a vertebrate cell type with multipotency by using the large out-of-sample mESC cell gene editing dataset (20). In parallel, we also compared the mechanistically informed MENTHU algorithm against inDelphi (23) and Lindel (21), two machine learning-based tools trained to predict DSB repair. As shown on Figure 3A, MENTHU and inDelphi offer distinct advantages over one another, at least on this large data set. MENTHU was able to identify close to twice as many PreMAs as inDelphi, amounting to around half of all available PreMAs in the dataset, though at a higher false positive rate. Even so, most of these false positives came from a slight overestimation of outcome frequencies (i.e. the observed repair outcome not reaching the required 50% minimum frequency for PreMA classification), and still displayed the predicted outcome sequence. This was also the case for the predicted true positives. This study shows how MENTHU performs satisfactorily in a different vertebrate cell type than zebrafish, and assuming this trend generalizes over multiple cell types, these results suggest that MENTHU could be a valuable tool for genome-wide gene discovery applications. The less sensitive inDelphi, on the other hand, does provide users with a ∼20% increase in PPV. This suggests that inDelphi could be useful as a PreMA confirmation tool for highly desirable DSBs. In contrast, Lindel correctly identified less than 1% of the available PreMAs in the data and therefore was not considered for the remaining PreMA comparative analyses.

Exploring the ROC curves for MENTHU and inDelphi (Figure 3B) provides a richer comparison between both tools. First, the inDelphi results appear marginally better overall (since curves closer to the upper left in an ROC plot are better), but note that this comparison is potentially biased in favor of inDelphi since the test set comprised data from the same type of mammalian mESC cells as the inDelphi training data (while MENTHU was trained on zebrafish cells). Also, the curves suggest that the performance differences noted above are mostly due to the specific prediction thresholds chosen for classification in the initial publications, since results for both models approximate each other by choosing different thresholds. Unfortunately, due to the nature of the inDelphi repair outcome predictions, choosing a different prediction threshold in their case is counterintuitive. The reason is that inDelphi predicts the frequencies of the different genetic outcomes per DSB directly. As such, claiming that a threshold different to 50% should be used to observe a single repair outcome 50% or more of the time is contradictory. Additionally, inDelphi does not currently enable users to modify the prediction threshold that gives rise to their predicted frequencies. In contrast, MENTHU provides the user the ability to filter out results below a user-specified MENTHU score, enabling users to choose this threshold to their liking (22). This paper aims to guide MENTHU users so they can fully take advantage of the ability to modulate sensitivity and specificity of their predictions.

Figure 4 suggests the existence of a 0–4 bp ∂ window to maximize PreMA repair outcomes. While the proportion of PreMAs decreases consistently within this window (Figure 4B), we observed a higher number of MMEJ events when ∂ ≥ 2 bp. Shifting the MENTHU ∂ requirement down to only include this 0–4 bp window (MENTHU@4) increased the prediction PPV and specificity without a large sacrifice in sensitivity. In our opinion, since PreMA targeting provides genome engineers and gene therapists with a method to better ensure experimental reproducibility, this small sensitivity trade-off is worth the 10% increase in PPV. The ROC curves displayed in Figure 5 display the classification performance of the original MENTHU (MENTHU@5), inDelphi, and the MENTHU-based Moon Rover and Moon Walker on the same out-of-sample test set. For all levels of specificity, Moon Rover and Moon Walker achieved a higher sensitivity than MENTHU and displayed performances virtually indistinguishable from each other (AUC, area under the curve, of 0.916 for both) and from that of inDelphi (AUC of 0.918). Moon Rover and Moon Walker, therefore, provide alternatives to MENTHU with performance levels comparable to inDelphi, without any of the conceptual issues that arise when customizing the prediction threshold.

Our results suggest that the addition of PreMA-targeting schemes to experimental pipelines using MENTHU or MENTHU-like tools is beneficial for both gene therapy and genome-wide reverse genetics applications. More specifically, we have shown that MENTHU was able to identify close to half of all available PreMAs in a large dataset. By looking at the MENTHU-predicted PreMA distribution of individual genes we also aimed to investigate if PreMA targeting would be useful at an intragenic scale, particularly for gene knockout experiments. It is worth noting that, in the case of generating out-of-frame mutations, insertion-based frameshifts may also provide alternative targeting options. In the dataset analysed, single majority insertions constituted a non-trivial ∼36% of all frameshift mutations, the rest being PreMAs. Therefore, we believe that there is significant value to pursuing insertion-based single majority outcomes, with inDelphi and Lindel providing two different avenues for such predictions. While Lindel correctly identified ∼33% of all single majority insertion frameshifts, inDelphi did not predict any. Thus, we propose to combine MENTHU PreMA predictions with Lindel insertion predictions (MENdel) to cover both deletion- and insertion-based single majority frameshifts. MENdel provided the largest coverage of single majority frameshifts without any major trade-offs.

By prospectively screening 54 vertebrate genes for PreMAs we estimated that, on average, four out of every five genes display at least one SpCas9-targetable PreMA within the first 30% of their coding sequences, (and close to nine in ten genes when considering MENdel or Cas12a PreMAs as well). Of note, these are likely underestimations, since we did not account for any DSBs derived from splice site targeting. Taken together, we believe that the systematic targeting of out-of-frame single majority outcomes to decrease the heterogeneity of the genotypic pool that results from DSB repair should be a viable strategy for almost all genes for the generation of knockouts, potentially accelerating gene discovery and gene therapy applications, and that tools like MENTHU, inDelphi and MENdel currently empower genome engineers to do so. It is worth noting that restricting frameshift analyses to single majority outcomes ignores the possibility of gene inactivation via the combination of all generated mutant alleles. Since frameshifts at different positions within a gene can result in varying levels of inactivation, we focused on single majority edits to enrich for experimental reproducibility. However, MENdel results output both the expected MENTHU MMEJ deletions as a function of μH competition (MENTHU Score) and the Lindel-predicted insertion frequencies, from which users can infer combined-allele frameshifts.

Another feature of interest of MENTHU is the hypothesis behind the algorithm. Firstly, MENTHU only predicts PreMAs when the μHs involved in repair are relatively close. This is due to the assumption that the kinetics of the DSB repair machinery would favor μH pairs that are physically closer to each other. The exponential decrease in PreMA fraction as a function of ∂ supports this assumption (Figure 4B). Secondly, the MENTHU score was designed as a measure of the competitiveness between the μH options the repair machinery has to bridge at a given DSB. Mathematically, the MENTHU score is the ratio between the Bae *et al.* (16) μH pattern scores of the top two predicted outcomes repaired by MMEJ (Figure 1B’’). These pattern scores can be interpreted as the ‘strength’ of a μH pair and were shown to correlate with the observed occurrence frequency of the corresponding MMEJ repair outcome by the authors. In addition to the proximity between the μHs used for repair, MENTHU requires the ratio of these ‘strengths’ to be 1.50 or above and interprets this scenario as a low competition state. In this case, the ‘strongest’ μH pair is ‘stronger’ enough than the other, which results in a higher propensity to be picked by the MMEJ machinery for repair. The success of MENTHU as a PreMA predictor supports this competition hypothesis, and potentially sheds some light into the function of the underlying biochemical mechanism. This is in contrast to the machine-learning based inDelphi which, albeit displaying comparable prediction levels to MENTHU, is difficult to interpret in terms of features or biological mechanisms, since the multiple hidden layers rapidly transform and integrate input features. That said, inDelphi predictions are a result of an ensemble of three machine learning models: two deep networks for deletions and a k-nearest neighbor scheme for insertions, and the authors include the result of the first two in the calculation of the latter, hinting at competition between deletions and insertions.

Inspired by the competition hypothesis, Moon Rover and Moon Walker employ the same two features that MENTHU uses for PreMA predictions. In Moon Rover, only the proximity criterion displayed a significant *p* value (*p* $ < 2\; \times {10^{ - 6}}$)), meaning that it is unlikely to have observed the results of the logistic regression by chance alone, while the MENTHU score did not show a significant *p* value (*p* = 0.468). In Moon Walker, the relative influence of each variable was calculated using the caret package in R by averaging the accuracy improvement made by each individual predictor variable at each decision split across all decision trees. According to Moon Walker, the proximity criterion was approximately 4 times more influential for accurate PreMA classification (∼80% to ∼20%). Thus, both Moon Rover and Moon Walker suggest that μH proximity is an important factor for PreMA prediction, and probably relevant to the underlying MMEJ repair biochemistry. Perhaps not as influential as proximity, the MENTHU score still improves predictions across all MENTHU-based classifiers, and future studies should investigate how to better quantify and measure μH competitiveness. The pattern scores that comprise the MENTHU score are metrics that aggregate μH length, GC content, and expected deletion length, and we are agnostic as to whether the pattern score is the best possible surrogate for μH ‘strength’. It is also likely that the features described above are not the only decisive factors in swaying the repair machinery to MMEJ preferentially, and we expect that the biochemical context surrounding the repair process to be heavily influential to factors such as cell cycle stage, DNA accessibility, and more.

Precise genome engineering technology is functionally a two-step process: the generation of a specific DSB and its subsequent repair. Currently, the efficient generation of consistent DSB repair outcomes remains an important bottleneck for precision gene editing, with traditional nuclease design yielding around a 10% chance for a PreMA reagent (15,23) (for reference, 10.4% of Allen *et al.* (20) gRNAs are PreMA reagents). MENTHU, inDelphi, and Lindel offer genome engineers better control over the second step, enabling researchers to generate more consistent genotypes for their gene editing experiments. Here, we show that MENTHU and inDelphi can identify large fractions (46% and 23%, respectively) of all available PreMA sites on an independent dataset with over 50% precision (PPV). We also introduce the novel workflow MENdel that combines MENTHU PreMA targeting with Lindel insertion predictions, and used it to identify DSBs likely to result in frameshift-inducing single majority outcomes for loss-of-function experiments. According to MENdel screens of 54 vertebrate genes, we estimate that close to 90% of genes (47/54) should possess at least one SpCas9-targetable single majority frameshift site. Around 80% of these (44/54 genes) are expected to be PreMAs (the remaining being due to 1bp insertions). Unlike inDelphi or Lindel, MENTHU allows the PreMA prediction of DSBs generated by nucleases other than SpCas9. According to our MENTHU estimates, considering Cas12a-targetable sites (TTTV PAM) in addition to SpCas9 increases the gene coverage for frameshift-inducible PreMAs to 87% (47/54). Taking the MENdel SpCas9 predictions together with the MENTHU Cas12a predictions showed brought the number of genes expected to have at least one knockout generating single majority outcome to over 90% (49/54). According to these estimates, screening for single majority sites represents a novel precision gene editing approach that facilitates consistent and reproducible outcomes for gene therapy and gene discovery applications.

## DATA AVAILABILITY

The DNA double-strand break repair data by Allen and collaborators is available at https://figshare.com/articles/processed_mutational_profiles/7312067. MENTHU is hosted at genesculpt.org/menthu. inDelphi is hosted at indelphi.giffordlab.mit.edu. Lindel is hosted at lindel.gs.washington.edu/Lindel/. MENdel is hosted at github.com/FriedbergLab/MENdel-command-line. ENSEMBL accession numbers for the CDS sequences screened for early loss-of-function PreMAs are included in Supplemental Table S1.

## Supplementary Material

gkaa1156_Supplemental_File
