# Supplementary material for: Deploying MMEJ using MENdel in precision gene editing applications for gene therapy and functional genomics
Source: Nucleic Acids Res. 2020 Dec 10;49(1):67–78. doi: 10.1093/nar/gkaa1156 (PMC7797032; doi:10.1093/nar/gkaa1156)
Supplement: gkaa1156_Supplemental_File [file gkaa1156_supplemental_file.doc]

**SUPPLEMENTAL TABLE 1**

| **Species** | **Transcript** | **Ensembl ID** | **CDS length (bp)** | **CDS length until frameshift (bp)** |
| --- | --- | --- | --- | --- |
| **Human** | AARS2-201 | ENST00000244571.5 | 2999 | 1000 |
|  | ATP5F1E-201 | ENST00000243997.8 | 197 | 182 |
|  | BCS1L-210 | ENST00000431802.5 | 1301 | 434 |
|  | COQ2-202 | ENST00000311469.9 | 1307 | 436 |
|  | COQ7-201 | ENST00000321998.10 | 695 | 182 |
|  | COX10-201 | ENST00000261643.8 | 1373 | 458 |
|  | COX4I2-201 | ENST00000376075.3 | 557 | 182 |
|  | COX6B1-201 | ENST00000246554.7 | 302 | 182 |
|  | CSF2-201 | ENST00000296871.4 | 476 | 182 |
|  | ERCC6-201 | ENST00000355832.10 | 4523 | 1508 |
|  | FXN-207 | ENST00000643639.1 | 674 | 182 |
|  | GJB2-203 | ENST00000645189.1 | 722 | 182 |
|  | KIT-201 | ENST00000288135.5 | 2972 | 991 |
|  | MCU-204 | ENST00000536019.5 | 950 | 317 |
|  | MICU1-201 | ENST00000361114.10 | 1472 | 491 |
|  | MICU2-201 | ENST00000382374.9 | 1346 | 449 |
|  | MTFMT-201 | ENST00000220058.9 | 1211 | 404 |
|  | NDUFA9-201 | ENST00000266544.10 | 1175 | 392 |
|  | NDUFAB1-205 | ENST00000570319.5 | 512 | 182 |
|  | NDUFAF6-202 | ENST00000396113.5 | 767 | 182 |
|  | NDUFS4-201 | ENST00000296684.10 | 569 | 182 |
|  | PDSS2-202 | ENST00000369037.9 | 1241 | 414 |
|  | SDHA-201 | ENST00000264932.11 | 2036 | 679 |
|  | SHH-201 | ENST00000297261.7 | 1430 | 477 |
|  | SMDT1-207 | ENST00000571525.3 | 365 | 182 |
|  | SURF1-201 | ENST00000371974.8 | 944 | 315 |
|  | TK2-201 | ENST00000299697.11 | 839 | 182 |
|  | TRAC-201 | ENST00000611116.2 | 426 | 182 |
|  | UQCRQ-203 | ENST00000378670.8 | 290 | 182 |
| **Zebrafish** | aars2-203 | ENSDART00000184440.1 | 1241 | 414 |
|  | bcs1l-201 | ENSDART00000022246.8 | 1304 | 435 |
|  | coq2-201 | ENSDART00000082797.6 | 1178 | 393 |
|  | coq7-201 | ENSDART00000090709.5 | 713 | 182 |
|  | cox10-201 | ENSDART00000048927.7 | 1385 | 462 |
|  | cox4i2-201 | ENSDART00000104487.5 | 575 | 182 |
|  | cox6b1-201 | ENSDART00000066506.5 | 302 | 182 |
|  | cx30.3-201 | ENSDART00000062669.6 | 845 | 182 |
|  | fxn-201 | ENSDART00000142577.2 | 644 | 182 |
|  | mcu-201 | ENSDART00000165259.2 | 1172 | 391 |
|  | micu1-202 | ENSDART00000132301.3 | 1511 | 504 |
|  | micu2-201 | ENSDART00000097677.5 | 1301 | 434 |
|  | mtfmt-201 | ENSDART00000053814.8 | 1214 | 405 |
|  | ndufa9a-201 | ENSDART00000019413.11 | 1184 | 395 |
|  | ndufab1b-201 | ENSDART00000022147.8 | 503 | 182 |
|  | ndufaf6-201 | ENSDART00000075718.6 | 1028 | 343 |
|  | ndufs4-201 | ENSDART00000051055.6 | 548 | 182 |
|  | pdss2-201 | ENSDART00000012232.10 | 1154 | 385 |
|  | pkd1a-201 | ENSDART00000039911.8 | 13082 | 4361 |
|  | sdha-201 | ENSDART00000015559.11 | 2027 | 676 |
|  | smdt1a-201 | ENSDART00000149709.2 | 375 | 182 |
|  | surf1-201 | ENSDART00000011337.8 | 971 | 324 |
|  | tk2-205 | ENSDART00000149186.3 | 710 | 182 |
|  | tmem70-201 | ENSDART00000109160.4 | 788 | 182 |
|  | tp53-201 | ENSDART00000051549.5 | 1166 | 389 |
|  | ttn.2-201 | ENSDART00000109099.4 | 22845 | 7615 |
|  | uqcrq-202 | ENSDART00000173082.2 | 290 | 182 |
